# A randomized controlled trial investigating the neurocognitive effects of Lacprodan® PL-20, a phospholipid-rich milk protein concentrate, in elderly participants with age-associated memory impairment: the Phospholipid Intervention for Cognitive Ageing Reversal (PLICAR): study protocol for a randomized controlled trial

**DOI:** 10.1186/1745-6215-14-404

**Published:** 2013-11-26

**Authors:** Andrew B Scholey, David A Camfield, Matthew E Hughes, Will Woods, Con K K Stough, David J White, Shakuntla V Gondalia, Pernille D Frederiksen

**Affiliations:** 1 Centre for Human Psychopharmacology, Swinburne University of Technology, Melbourne 3122, Victoria, Australia; 2 Brain & Psychological Sciences Research Centre, Swinburne University of Technology, Melbourne, Australia; 3 Arla Food Ingredients, P/S Sønderhøj 14, 8260, Viby J, Denmark

## Abstract

**Background:**

Age-related cognitive decline (ARCD) is of major societal concern in an ageing population, with the development of dietary supplements providing a promising avenue for amelioration of associated deficits. Despite initial interest in the use of phospholipids (PLs) for ARCD, in recent years there has been a hiatus in such research. Because of safety concerns regarding PLs derived from bovine cortex, and the equivocal efficacy of soybean-derived PLs, there is an important need for the development of new PL alternatives. Phospholipids derived from milk proteins represent one potential candidate treatment.

**Methods:**

In order to reduce the effects of age-associated memory impairment (AAMI) the Phospholipid Intervention for Cognitive Ageing Reversal (PLICAR) was developed to test the efficacy of a milk protein concentrate rich in natural, non-synthetic milk phospholipids (Lacprodan® PL-20). PLICAR is a randomized, double-blind, placebo-controlled parallel-groups study where 150 (N = 50/group) AAMI participants aged > 55 years will be randomized to receive a daily supplement of Lacprodan® PL-20 or one of two placebos (phospholipid-free milk protein concentrate or inert rice starch) over a 6-month (180-day) period. Participants will undergo testing at baseline, 90 days and 180 days. The primary outcome is a composite memory score from the Rey Auditory Verbal Learning Test. Secondary outcomes include cognitive (verbal learning, working memory, prospective and retrospective memory, processing speed and attention), mood (depression, anxiety, stress and visual analogue scales), cardiovascular (blood pressure, blood velocity and pulse wave pressure), gastrointestinal microbiota and biochemical measures (oxidative stress, inflammation, B vitamins and Homocysteine, glucoregulation and serum choline). Allelic differences in the Apolipoprotein E and (APOE) and *Methylenetetrahydrofolate reductase* (MTHFR) gene will be included for subgroup analysis. A subset (N = 60; 20/group)) will undergo neuroimaging using functional magnetic resonance imaging (fMRI) and magnetoencephalography (MEG) in order to further explore *in vivo* central mechanisms of action of Lacprodan® PL-20. This study will enable evaluation of the efficacy of milk-derived phospholipids for AAMI, and their mechanisms of action.

**Trial Registration:**

The trial is jointly funded by Arla Foods and Swinburne University of Technology, currently recruiting and is registered on the Australian New Zealand Clinical Trials Registry as ACTRN12613000347763.

## Background

Cognitive deficits including learning and memory impairment are one of the most prominent and debilitating consequences of normal and pathological ageing in humans. A consistent finding in research on ageing and cognition is that performance across various tests of memory is lower with increased age. Meta-analyses of cross-sectional and longitudinal datasets have demonstrated approximately 40 to 60% decline in cognitive speed at age 80 compared to age 20 years in non-demented adults [1]. These cognitive deficits are of considerable concern to elderly individuals, with up to 50% of adults aged 64 years or older reporting difficulties with their memory [2]. There is an increasing awareness of the possibility that dietary modification can alter the course of age-related cognitive decline.

Frequent dairy food intake is associated with better cognitive function, although the exact underlying mechanisms are yet to be determined. The positive correlation between increased dairy intake and cognitive function seems partly to be a result of counteracting metabolic syndrome, by reducing cardiovascular (CV) risk factors such as type 2 diabetes, hypertension, obesity and hyperlipedimia [3-5]. In a recent paper by Crichton *et al*. [6], the authors support this link and in addition argue that apart from counteracting the CV risk factors, dairy consumption provides additional benefits in relation to cognitive function (for example, after adjusting for CV disease, lifestyle and dietary factors). Recent advances in dairy manufacturing, which enable the enrichment of phospholipid components in milk also offer a promising new avenue for the treatment of age-related cognitive decline.

### Phospholipids and the amelioration of cognitive decline

Bovine milk contains a vast range of phospholipids and complex lipids, with important biological functions. Phospholipids are substances with both a hydrophilic (water-liking) head and hydrophobic (water-repellent) tail segment which are major building blocks for cellular membranes, including neuronal cells, where they arrange themselves into lipid bilayers [7]. Relevant phospholipids in relation to cognitive performance include phosphatidylserine (PS) and phosphatidylcholine (PC) as well as the related substances sphingomyelin and the sialic-acid-containing gangliosides. Pharmacokinetic studies of PS have revealed good bioavailability when consumed orally. Following ingestion, the headgroup is absorbed intact through the intestinal wall into the bloodstream, while the fatty-acid tail segments at position 2 are often removed and later re-added. After crossing the blood–brain barrier the fatty acid at position 2 is typically occupied by either oleic (C18:1) or docosahexaenoic acid (DHA, C22:6) in the brain [8,9].

#### Phosphatidylserine (PS)

PS is a naturally occurring membrane phospholipid that is found in high concentrations in brain tissue, where it comprises 10 to 20% of the total phospholipid pool [10]. PS plays an important role in a host of cellular functions including mitochondrial membrane integrity, presynaptic neurotransmitter release, postsynaptic receptor activity and activation of protein kinase C in memory formation [7,11]. PS enhances the activities of membrane-bound enzymes involved in signal transduction [12] and plays a key role in the biosynthesis and release of several neurotransmitters, including acetylcholine [13,14], norepinephrine [15], serotonin and dopamine [16]. PS has also been found to elevate glucose metabolism [17].

In relation to the cholinergic system, PS has been found to restore age-related decreases in choline-acetyltransferase-positive neurons [18] as well as densities of muscarinic and N-methyl-D-aspartate (NMDA) receptors [19,20]. Increases in nerve growth factor (NGF)-receptor density have been reported in aged animals following PS supplementation, as well as increases in neuronal numbers and size [21]. Other beneficial effects of PS supplementation include protection from cell death [22] due to increased membrane fluidity [23] as well as anti-inflammatory and antioxidant effects [24]. Behavioural animal experiments using PS have provided evidence of improvements in spatial memory [25], retention of passive avoidance [15], exploration and memory retrieval [26] as well as improvement of avoidance performance [27]. Prevention of scopolamine-induced learning deficits have also been reported [28-31], together with an attenuation of memory deficits associated with reserpine-induced catecholamine depletion [32].

PS is the most studied of the phospholipids in regards to human clinical trials of dementia and cognitive decline. Five double-blind randomized trials have been conducted using PS in Alzheimer’s disease (AD) [33-37]. Clinical global impressions of change and activities of daily living were found to be improved with daily doses of 200 to 300 mg up to six months. In milder cases of AD, significant improvements to concentration, learning and memory for names, locations and recent events were also observed [7,11]. In the largest of these studies involving 494 elderly patients with moderate to severe cognitive decline, Cenacchi *et al*. [37] reported improvements to memory and learning following 300 mg PS/day for 6 months.

In elderly populations with mild cognitive impairment and age-associated memory impairment (AAMI), PS has also been found to be effective in ameliorating cognitive declines. Crook *et al*. [38] administered 300 mg/day PS to 149 elderly patients (aged 50 to 75 years) with age-associated memory impairment for 12 weeks, and observed significant improvements in performance tests of learning and recall abilities such as name-face matching. In a multi-centre trial by Villardita *et al*. [39], 300 mg/day PS versus placebo was administered to 170 elderly patients for 90 days. Significant improvements in attention, concentration and short-term memory were found in those receiving PS. These earlier trials were conducted using PS derived from bovine cortex, but more recently trials have also been conducted using soybean (SB)-derived PS due to concerns over bovine spongiform encephalopathy. The results in relation to SB-PS have been more mixed, with Jorissen *et al*. [40] reporting no significant effects on learning and memory, whereas more recently Vakhaporva *et al*. [41] and Kato-Kataoka *et al*. [42] reported significant improvements to verbal learning following ≥3 months supplementation. An intriguing study by Hellhammer *et al*. [43] using the same milk-derived phospholipids as the current study (PL-20) also reported improvements to working memory function following 3 weeks supplementation in healthy adults aged 30 to 55 years.

#### Phosphatidylcholine (PC)

Choline is an essential nutrient critically needed for synthesis of the neurotransmitter acetylcholine; important in brain functions, such as memory and mood, but also important in skeletal-muscle control, heart rate and breathing. Numerous animal studies demonstrate that choline is necessary for normal development of the memory function and sub-optimal dietary intake of choline by the pregnant mother and later by the infant and child directly affects brain development and results in permanent changes in brain function (Zeisel *et al*. 1991). Choline or its metabolites, are also needed for the structural integrity and signaling functions of cell membranes; it is the major source of methyl-groups in the diet (one of choline’s metabolites, betaine, participates in the methylation of homocysteine (HCy) to form methionine), and it directly affects cholinergic neurotransmission, transmembrane signaling and lipid transport/metabolism [44,45]. Nagata *et al*. [46] documented recently that dietary supplements of PhosChol compounds (1,2-dilinoleoyl-sn-glycero-3-phosphocholine; DL-PC and 1-palmitoyl-2-oleoyl-sn-glycero-3-phosphocholine; PO-PC) ) enhanced the memory and learning ability in the elderly in the Mini Mental State Examination (MMSE). In another placebo-controlled clinical trial, Ladd *et al*. [47] found that the supplementation of SB-PC in normal college students lead to an improvement in explicit memory function due to increased choline supply and improved cholinergic function.

A poor folate status is associated with cognitive decline and dementia in older adults [48]. Although impaired brain methylation activity and HCy toxicity are widely thought to account for this association, how folate deficiency impairs cognition is still uncertain. Troen *et al*. [49] found a correspondence of cognitive outcomes to changes in brain membrane PC content (in rats), which suggests that altered PC and possibly choline metabolism might contribute to the manifestation of folate deficiency-related cognitive dysfunction. Up to 50% of older people have been reported to have folate deficiency, with higher levels in those institutionalized [50]. For this reason, there is a sound theoretical reasoning to speculate on a positive beneficial outcome in relation to cognition and boosting memory with choline and choline-containing compounds [44].

Elevated total homocysteine (tHcy), a risk factor for many chronic diseases including cognitive decline, can be remethylated to methionine by folate [51]. Alternatively, tHcy can be metabolized by other 1-carbon nutrients, that is, betaine and its precursor, choline. Elias *et al*. [48] reported that tHcy levels are inversely associated with visual-spatial organization, working memory, scanning-tracking, and abstract reasoning. Chiuve *et al*. [52] assessed the association between the dietary intakes of betaine and choline and the concentration of tHcy. They found the total choline + betaine intake to be inversely associated with tHcy. In a double-blind, placebo-controlled clinical trial Olthof *et al*. [53] investigated the supplementation with soybean PC (PhosChol) on homocysteine plasma concentrations in men with mildly elevated levels. They found that PC was able to reduce homocysteine levels, thus supporting the link between dietary intake of PC and homocysteine plasma levels.

#### Gangliosides

Brain content of specific gangliosides (for example, GM1) has been documented to decrease with age, and a low GM1 content has been observed in the brains of patients with AD [54]. Exogenously administered gangliosides have been shown to exhibit neurotrophic action, and to increase the release of brain-derived neurotrophic factor (BDNF) *in vitro*[55]. Experimental data have shown that gangliosides, and in particular, GM1, exhibit properties similar to the neurotrophins. The neurotrophins promote neurogenesis, which is essential for specific cognitive functions that decline in some neurological disorders and in ageing [56]. A systemic administration of GM1 in rats ameliorated the age-related decreased activity of choline acetyl transferase and choline uptake in the brain of aged rats as well as improved spatial learning and memory tasks in the aged rats [57]. Dietary gangliosides increase total brain-ganglioside content in rats [58].

#### Sphingomyelin (SM)

The myelin content of the brain decreases with age and the age-related slowing in cognitive processing speed is associated with myelin integrity in a very healthy elderly population [59]. Dietary bovine SM contributes to central nervous system (CNS) myelination [60]. Sphingomyelin is also a source of choline. Clinical trials are yet to be conducted in order to assess the *in vivo* neurocognitive effects of SM in humans.

### Methods/Design

Phospholipid intervention for cognitive ageing reversal (PLICAR) is a randomized, double-blind, placebo-controlled, three-arm, stratified parallel-groups clinical trial with participants randomized to receive a minimum daily dosage of 2.7 g phospholipids (and minimum daily dose of 300 mg/day PS) from Lacprodan® PL-20 or one of two placebos (milk protein concentration or rice starch) over a 180-day (6-month) period. The maximum daily dose of Lacprodan® PL-20 will be 16 g/day. Participants will be stratified according to age, IQ using Raven’s Progressive Matrices (RPM) [61] and baseline score on the Wechlser Memory Scale-Revised (WMS-R) [62].

### Aims and study hypotheses

The primary objective of the current study is to evaluate the chronic effects of daily Lacprodan® PL-20 supplementation on cognitive performance in a healthy elderly population with age-associated memory impairment (AAMI). The secondary objectives of the study are to investigate mood and CV effects of Lacprodan® PL-20 as well as the *in vivo* mechanisms of action by which Lacprodan® PL-20 may improve cognitive function. To this end, a range of measurement modalities will be employed including assessments of CV function, blood biomarkers, gastrointestinal (GI) microbiota, pharmacogenomics and brain activity assessed by functional magnetic resonance imaging (fMRI), magnetoencephalography (MEG) and diffusion tensor imaging (DTI).

Performance on the various outcome measures for participants receiving Lacprodan® PL-20 will be compared to the performance of participants receiving (i) an inert placebo powder made of rice starch, and (ii) a milk-protein concentrate without phospholipids that has been matched for protein content and total calories. The reason/rationale for including two comparator treatments (rather than one) is the opportunity to address separate research questions. The first comparison (to inert placebo) addresses the research question of whether Lacprodan® PL-20 as a whole has benefits for cognitive function and other secondary outcomes. The second comparison to a milk-protein concentrate without phospholipids addresses a more specific research question regarding whether benefits to cognition and other outcomes associated with Lacprodan® PL-20 can be attributed specifically to the phospholipid content in the formula. In consideration of previous research demonstrating cognitive benefits associated with dairy components other than phospholipids [3], the inclusion of a third treatment arm was deemed necessary in order to properly delineate the effects of phospholipids versus other dairy components present in Lacprodan® PL-20.

On the basis of previous human clinical studies with bovine and plant-derived phospholipids, it is hypothesised that in comparison to the inert placebo, Lacprodan® PL-20 supplementation over 180 days will result in significant improvements on our primary variable, namely a composite memory score computed from the Rey’s Verbal Learning Test (RVLT). We are also exploring the possibly that the treatment may benefit other elements of cognitive performance (including processing speed and global functioning). A second hypothesis is that cognitive benefits of a lesser magnitude will be observed when comparing supplementation with Lacprodan® PL-20 with milk protein concentrate (MPC) without phospholipids placebo over 180 days. Additionally we are exploring the effects of Lacprodan® PL-20 on a number of central, CV and GI biomarkers.

### Study site

PLICAR will be conducted at the Centre for Human Psychopharmacology, Swinburne University of Technology, Melbourne, Australia.

### Participants

A total of 150 healthy elderly participants (≥55 years) with AAMI will take part in the study. AAMI is defined on the basis of criteria first outlined by Crook *et al*. [63,64]: (i) a score >25 on the Memory Complaint Questionnaire (MAC-Q [64]) and (ii) a score ≤1 standard deviation below the mean for healthy young adults on the paired associates test from the WMS-R [62]. Participants will be excluded from participation if they are currently diagnosed with dementia and/or score <24 on the MMSE [65]; have a neurological, cardiac, endocrine, GI or bleeding disorder; have a psychiatric illness, including moderate-to-severe depression, as defined as a score ≥20 on the Beck Depression Inventory II (BDI-II) [66]; have a current or previous history of alcoholism and/or substance abuse; have a known or suspected allergy to cow’s milk and/or lactose intolerance; are a current smoker, or are not fluent in the English language. To be eligible, participants also cannot be currently taking any medications or herbal/dietary supplements with known cognitive effects. The study is jointly funded by Arla Foods (Denmark) and Swinburne University of Technology. It was ethically approved by the Swinburne University Human Research Ethics Committee (project number 2012/294) and all participants will provide written informed consent. The trial has been registered with the Australian and New Zealand Clinical Trials Registry (ACTRN12613000347763).

### Procedure

**Figure 1:**
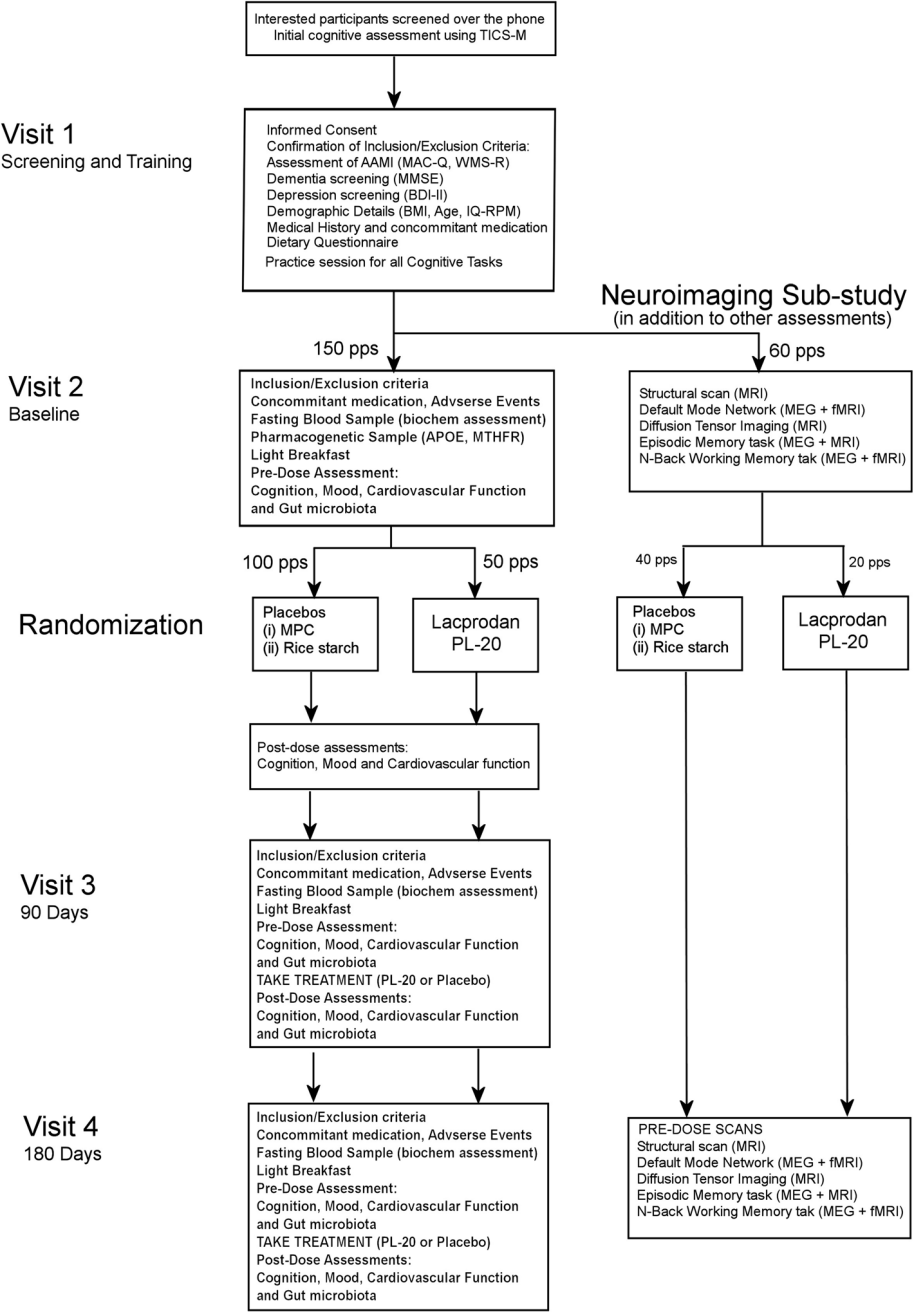
**Phospholipid intervention for cognitive ageing reversal (PLICAR) protocol flow diagram.** AAMI, age-associated memory impairment; MAC-Q, Memory Complaint Questionnaire; MMSE, Mini Mental State Examination; BDI-II, Beck Depression Inventory II; BMI, body mass index; RPM, Raven’s Progressive Matrices; APOE, Apoliprotein E; MTHFR, Methylenetetrahydrofolate reductase; MRI, magnetic resonance imaging; MEG, magnetoencephalography; MPC, milk protein concentrate; TICS-M telephone interview for cognitive status modified.

Eligible participants are required to attend four testing sessions. An overview of the testing sessions is provided in the clinical trial flow chart (Figure 1).

#### Visit 1 (screening/practice)

During the first visit, voluntary written informed consent is obtained from all participants. Then the participants are further screened for eligibility and administered the MMSE, MAC-Q, WMS-R and BDI-II. A detailed medical history is also taken, a dietary questionnaire is administered [67,68] and demographic information is collected, which includes body mass index (BMI), age, educational background and general intelligence (as measured by RPM). All eligible participants are then required to complete practice versions of all the cognitive outcome measures to be used in the study.

#### Visit 2 (Baseline)

The second visit will be scheduled for one week following the screening/practice visit. In preparation for the baseline visit, all participants will be required to collect and store their faecal sample (as per the procedure provided in the faecal sample collection kit) a day before the actual visit. The faecal sample for GI microbiota analysis will be deposited at the Centre for Human Psychopharmacology, Swinburne University, when the participant comes for the baseline visit. The faecal samples will be stored at -80°C until further analysis. Also in preparation, participants will be required to fast from 10 pm the night before. A fasting blood sample will then be taken in order to assess the baseline biochemical measures, together with a separate blood sample, which will be used for Apolipoprotein E (APOE) and *Methylenetetrahydrofolate reductas*e (*MTHFR*) genotyping. Following the blood samples, participants will eat a light breakfast. Half an hour after breakfast, participants will be required to complete all pre-dose cognitive, mood and CV measures. Participants will then be randomized to their treatment group (Lacprodan PL-20® or MPC or rice starch) and consume their first sachet of study treatment dissolved in water. A lunch break of 90 minutes will then follow, before post dose testing on all cognitive, mood and cardiovascular measures. At the conclusion of the baseline study visit, participants will be provided with enough study treatment for the next 90 days.

#### Visits 3 and 4 (90 and 180 days)

The schedule of events for the 90-day and 180-day visits is identical to that for the baseline visit. On the day of each study visit participants will be required to wait until the completion of pre-dose assessments before consuming their daily study treatment.

#### Neuroimaging sub-study

Participants involved in the neuroimaging sub-study will be required to attend two additional sessions, the first in the week preceding their baseline visit and the second in the week preceding their 180-day visit (see Figure 1). In the second visit they will be required to consume their study treatment as usual in the morning before the scans. This is due to the fact that the neuroimaging sub-study is only concerned with the chronic effects of PL-20 on brain function.

### Sample size

Based on the reviewed literature, we predict a small-medium effect size (*f* = 0.14) on the primary variable. The sample size for this study is 150 participants, with 50 participants in each treatment group (Lacprodan® PL-20, MPC or rice starch). Allowing for a 20% drop-out rate over the course of the 180-day testing period, this will give 80% power to detect significant treatment × time interactions from baseline to 180 days for the primary outcome (calculated using G*Power 3.1, with α = 0.05 and *r* = 0.5 for the correlation between repeated measures).

### Treatments

Lacprodan® PL-20, a powdered MPC rich in phospholipids, is manufactured by Arla Foods Ingredients Group P/S, Viby J, Denmark. The content of individual phospholipids in Lacprodan® PL-20 is displayed in Table 1.

**Table 1:** Phospholipid composition of Lacprodan® PL-20 by percentage and daily dose

	**Percentage (%)**	**Minimum dose per day**
Sphingomyelin	4.3	688 mg
Phosphatidyl choline (PC)	4.3	688 mg
Phosphatidyl serine (PS)	1.9	304 mg
Phosphatidyl ethanolamine (PE)	3.5	560 mg
Phosphatidyl inositol (PI)	1.3	208 mg
Ganglioside and others	0.7	112 mg

Lacprodan® PL-20 will be administered orally at a maximum dose of 16 g/day, providing minimum daily dosages of 2.7 g PL and 300 mg PS. The powder is dissolved in 150 to 200 mL water and drunk once per day with breakfast. Two placebo treatments will also be administered: (i) an inert placebo consisting of rice starch (20 g/day), and (ii) an MPC without phospholipids (Arla Foods); (12 g/day). Both placebo treatments are also administered orally as powders dissolved in ≤250 mL water and matched to Lacprodan® PL-20 for colour and taste in order to ensure treatment blinding.

### Randomization and safety

Randomization of participants to treatment groups will be determined by random allocation. For the neuroimaging sub-study 60 randomization numbers will be set aside, which correspond to 10 female participants receiving Lacprodan® PL-20, 10 receiving MPC and 10 receiving rice starch placebo, and 10 male participants receiving Lacprodan® PL-20, 10 receiving MPC and 10 receiving rice starch placebo. Blinding for both the main study as well as the neuroimaging sub-study will be conducted by an independent staff member at the Centre for Human Psychopharmacology, who is outside of the project, to code the treatments, and maintain the key to this code until data collection is completed. All potential adverse events will be monitored throughout the trial, with oversight from the Swinburne University of Technology Human Research Ethics committee.

### Primary outcomes

The primary study outcome is the effect of Lacprodan® PL-20 supplementation on memory as measured using a compound score from the RVLT [69,70], similar to that used previously [71]. The memory score will be derived using the formula:

$$\begin{array}{l} \left(Z_{15‒\mathrm{Word}\;\mathrm{Learning}\;\mathrm{test}‘\mathrm{total}\;\mathrm{immediate}\;\mathrm{recall}}’\right)\\ \;+Z_{15‒\mathrm{Word}\;\mathrm{Learning}\;\mathrm{Test}‘\mathrm{maximum}\;\mathrm{immediate}\;\mathrm{recall}’}\\ \left(\;+Z_{15‒\mathrm{Word}\;\mathrm{Learning}\;\mathrm{Test}‘\mathrm{delayed}\;\mathrm{recall}’}\right)/3. \end{array}$$

The RVLT is a test of verbal learning and memory that has a long history of use both in the assessment of clinical memory disturbances as well as cognitive decline associated with normal ageing [70]. Verbal learning, as measured by the RVLT and similar tests, has previously been found to be sensitive to the effects of phospholipid interventions in AAMI populations [39,41]. Similarly, verbal memory has also been found to be sensitive to other nutraceutical interventions such as *Bacopa monnieri*[72] and folic acid [71] in elderly populations. For these reasons, the inclusion of the RVLT will enable direct comparison of the efficacy of the milk-derived phospholipids present in Lacprodan® PL-20 to previous cognitive intervention studies in the elderly.

### Secondary outcomes

A range of secondary outcomes will be used, encompassing cognitive performance, mood, CV, GI microbiota, biochemical, genetic and brain imaging modalities. Secondary outcomes will include other elements of cognitive performance as measured by a battery of well-validated and highly sensitive cognitive tests. Traditional paper-and-pencil neuropsychological tests as well as computerized tasks have been included. These tests will be implemented at baseline, 90 days and 180 days (pre-dose) in order to capture chronic effects. This battery will consist of the MMSE [65], the Prospective and Retrospective Memory Questionnaire (PRMQ) [73], RVLT [69,70], Spatial Working memory and Contextual Memory tasks from the Swinburne University Computerized Cognitive Ageing Battery (SUCCAB [74]), rapid visual information processing (RVIP), serial 3 s and 7 s subtraction and the Hick’s reaction time paradigm. In order to capture potential acute cognitive effects associated with Lacprodan® PL-20 supplementation, the SUCCAB tests, RVIP, serial 3 s and 7 s subtraction and Hick’s reaction time will also be administered 90 minutes post dose.

The MMSE [65] is a global measure of cognitive function that has been used extensively both as a diagnostic tool for dementia screening as well as a cognitive outcome measure for gauging the efficacy of chronic nutraceutical interventions in elderly participants, for example [71,75,76]. The MMSE was included in the current study due to its widespread use in previous research; however problems with ceiling effects and insensitivity to change amongst high-functioning individuals have been previously well-documented [77].

The PRMQ [73] is a self-report instrument which provides a measure of retrospective as well as prospective memory slips in everyday life. The PRMQ was included in the current study in order to provide an ecologically valid measure of typical memory complaints that may be of concern to elderly individuals. Although most memory complaint questionnaires focus exclusively on failures to remember previous information (retrospective memory), the PRMQ is unique in that it additionally provides a measure of prospective memory failures, which are failures relating to tasks that need to be completed at a certain time (for example, remembering to turn up to an appointment on time) [73,78].

In addition to the use of traditional psychometric tests, the importance of including computerized tests that may accurately gauge the speed of well-differentiated cognitive functions has emerged in recent years [79]. In the current study the spatial working memory and contextual memory tasks from the SUCCAB have been included due to high degrees of sensitivity to the effects of ageing, as measured using response times [74]. Similarly, significant reductions in response times on both of these tasks have previously been reported in older participants following chronic nutraceutical interventions [80,81]. For the assessment of processing speed, the highly sensitive Hick reaction time paradigm [82] will be used. For the assessment of cognitive function during increased demand, serial 3 s and serial 7 s subtraction will be assessed together with the RVIP computerized measure of sustained attention. Previous research from our laboratory has found the serial subtraction and RVIP tasks to be particularly sensitive to the acute effects of nutraceutical interventions [83-86].

#### Mood

There is evidence to suggest that phospholipid supplementation may have a positive effect on chronic stress as well as mood. Mood improvements have been previously reported in a double-blind trial of PS in depressed patients [87]. A number of studies have also demonstrated that phospholipids may have anti-stress effects, as demonstrated by lowered levels of adrenocorticotropic hormone (ACTH), reduction in perceived stress ratings in response to acute stress [88] and reduced cortisol release in response to acute stress [89,90]. In relation to milk-based phospholipids, it was recently demonstrated that chronic supplementation may lead to increased morning cortisol availability in chronically stressed men [91] as well as a blunting of self-report stress ratings in response to an acute stressor [43].

Chronic changes in mood over the course of the trial will be assessed using the Depression, Anxiety and Stress Scale (DASS) [92], the Profile of Mood States (POMS) [93] and Bond-Lader visual analogue mood scales [94]. These measures will be administered at baseline, 90 and 180 days pre-dose. The Bond-Lader scales will additionally be administered post dose at each study visit in order to capture potential acute mood effects associated with Lacprodan® PL-20 treatment. The Bond-Lader scales have previously been used by our group in a wide range of acute and chronic intervention studies, and have been found to display excellent sensitivity to subtle affective changes.

#### Cardiovascular assessment

There is evidence to suggest that increases in arterial stiffness with ageing, which are reflected in measures of blood flow velocity, may be a contributing factor in cognitive decline [95,96]. Previous research suggests that milk proteins may increase insulin secretion, as well as help to reduce blood pressure and plasma cholesterol levels [3,97]. Further, high levels of B12 present in milk may also help to lower HCy levels, which are a contributing factor to CV disease [98]. In light of the fact that Lacprodan® PL-20 is an MPC, it could be argued that chronic supplementation may have a positive influence on CV function. By this reasoning, the inclusion of CV parameters in the current study will enable exploration of whether improvements to CV function are a mechanism by which with Lacprodan® PL-20 may achieve cognitive benefits.

CV function will be assessed using brachial blood pressure, aortic blood pressure, carotid-femoral pulse wave velocity (PWV) as well as blood flow velocity in the medial carotid artery and the common carotid artery (CCA). Brachial blood pressure will be calculated with the participant seated and following a five-minute rest period using a clinically validated automated sphygmomanometer. Aortic blood pressure, pulse pressure and PWV (all aspects of arterial stiffness and CV pressures) will be measured non-invasively using the SphygmoCor device. Applanation tonometry of the radial artery will be used to estimate aortic pressures and wave reflections, and applanation of the carotid and femoral arteries will be used to measure PWV. A non-invasive transcranial Doppler system will be used to record middle cerebral artery (MCA) blood velocity by placing a sensor close to the participant’s ear and common carotid artery (CCA) blood velocity will be recorded by placing a hand-held sensor at the base of the participant’s neck. All CV measures will be assessed pre- and post dose at the baseline, 90-day and 180-day study visits in order to capture both acute and chronic effects associated with Lacprodan® PL-20 supplementation.

#### Gastrointestinal microbiota

In recent years research in the field of GI microbiota has caught major interest. Research is suggesting that modifications in the composition of the GI microbiota influence normal physiological functions and contribute to diseases ranging from inflammation to diabetes. Collectively studies now indicate that the gut microbiota also communicates with the CNS possibly through immune, neural and endocrine pathways, and by these means influences gut-brain communication, brain function and even behaviour [99-101]. Studies on germ-free animals and animals exposed to pathogenic bacterial infections, probiotic bacteria or antibiotics, suggest a role of GI microbiota in the regulation of cognition, anxiety and mood [101-103]. Moreover GI microbiota perform many important functions like protection, immune development and metabolism, which together have an enormous effect on host nutrition and health condition [104-106]. Previous studies suggest that human and bovine milk proteins prevent the adhesion and colonisation of pathogenic bacteria in the GI tract [107-109] and promote the growth of beneficial bacteria [110]. As knowing that GI microbiota have several physiological functions in the human health condition, it can be influenced by Lacprodan® PL-20 milk protein supplementation. Therefore, it will be valuable to study the GI microbiota at different time points across the clinical trial to identify the effect of Lacprodan® PL-20 on indigenes microbial community.

Faecal samples will be collected for the GI microbiota analysis at baseline, 90 days and 180 days to explore the possible effect of Lacprodan® PL-20 on the microbial composition. The microbiota analysis will be carried out by utilising deep next-generation shotgun sequencing [111] of DNA extracted from collected faecal samples. This analysis will provide insight into GI microbiota of the ageing population and also functional characterisation will provide understanding of the potential mechanism by which Lacprodan® PL-20 may influence age-related cognitive decline (ARCD).

#### Biochemical assessment

Haematological testing will be conducted at baseline, 90 days and 180 days in order to further investigate possible mechanisms by which Lacprodan® PL-20 may influence cognitive decline. These measures have been chosen on the basis of current aetiological understanding of brain ageing as well as proposed *in vivo* actions of Lacprodan® PL-20 constituents.

Previous research has demonstrated that administration of PC, a major phospholipid component of Lacprodan® PL-20, can increase the plasma choline as well as brain acetylcholine (Ach) supply [112,113]. Further, elevated levels of the neurotoxic substance HCy have been found to be a risk factor for cognitive decline [48,114-116]. Improved HCy levels have been found to result from increased intake of PC and choline [52,53]. Vitamin B12, which is present in high quantities in dairy products [117], has also been found to be effective in reducing HCy in elderly populations [118]. For these reasons plasma choline as well as B Vitamins and HCy levels will be monitored throughout the study.

Other major contributors to brain ageing are oxidative stress [119,120] and inflammation [121]. The phospholipids PC and PS have both been found to display anti-inflammatory and antioxidant properties, inhibiting microglial activation as well as superoxide and nitric oxide production [122]. Endogenous antioxidant glutathione (GSH) is the most abundant antioxidant in human cells, which plays a central role in defence from oxidative stress [123]. Under normal physiological conditions the ratio of reduced GSH to oxidized glutathione (glutathione disulphide, GSSG) is as high as 100:1. However, in cases of increased oxidative stress the ratio changes due to increased levels of GSSG or decreased levels of reduced GSH. For this reason peripheral blood levels of GSH as well as the ratio of GSH/GSSG in the blood are good measures of oxidative stress, and have been found to be altered in patients with mild cognitive impairment and AD [124]. Another widely-used biomarker of oxidative stress is F2-isoprostane, which is an excellent *in vivo* measure of lipid peroxidation [125]. Peripheral blood plasma levels of F2 isoprostane have been found to be significantly elevated in mild cognitive impairment [126]. A previous study from our laboratory in elderly participants found plasma F2 isoprostane levels to significantly decline following a 3-month intervention with the antioxidant Pycnogenol [127]. In addition to these measures of oxidative stress, serum measures of inflammation will be provided using the following inflammatory biomarkers: TNF-α, IL-1β, IL-6 and C-reactive protein (CRP). Peripheral levels of these inflammatory biomarkers have previously been found to be elevated in cases of mild cognitive impairment and AD in comparison to healthy age-matched controls [128,129].

#### Genetic

A separate blood sample will be collected pre-randomization for the analysis of single nucleotide polymorphisms (SNP) in the *APOE* and the *MTHFR* genes. The *APOE*-ϵ4 allele has been found to be associated with an increased risk of developing AD as well as cognitive decline in normal elderly [130,131]. Testing for allelic differences in the *APOE* gene was included in the current study in order to determine whether these genetic differences may affect the efficacy of Lacprodan® PL-20 as a treatment for AAMI. MTHR is an important enzyme involved in the metabolism of HCy. The *MTHFR* 677 T allele is associated with reduced enzymatic activity, which results in decreased serum and plasma levels of folate as well as increased plasma levels of HCy [132]. In consideration of the relationship between levels of phospholipids, vitamin B12 and HCy [53,118], genetic testing for allelic differences in the *MTHFR* gene was included in the current study in order to assess whether this may also affect the efficacy of Lacprodan® PL-20 as an AAMI treatment.

#### Neuroimaging

Neuroimaging with fMRI and MEG will be conducted in a subset of 60 participants in order to further explore the *in vivo* mechanisms of action of Lacprodan® PL-20 in the brain. Previous neuroimaging studies using PS supplementation in AD have been conducted using electroencephalography (EEG) as well as positron emission tomography (PET) [133-135]. PET results revealed that for the PS group there was increased glucose metabolism during a visual recognition task across a number of brain regions, most notably the temperoparietal regions [133]. However, to date no further neuroimaging studies have been conducted using phospholipid interventions, and to the best of our knowledge none have been conducted using MRI or MEG.

In the current study structural and functional MRI scans will be acquired using a Siemens 3 Tesla Tim Trio MRI scanner (Erlangen, Germany), located at the Centre for Human Psychopharmacology, Swinburne University of Technology. During the initial scan, a structural image will be obtained for each participant and used as a reference point for further functional scans. Scanning for DTI analysis, a measure of white matter integrity, will also be conducted. Following DTI there will be scanning in a resting state in order to assess activity in the default mode network (DMN) for approximately 6 minutes. Additional analysis of cell membrane fluidity will also be conducted by using the T2 signal timing information (relaxometry) while in a resting state. Changes in the blood oxygenation-level dependent (BOLD) signal will also be analysed while participants complete in-scanner versions of verbal episodic memory (approximately 20 minutes) and N-Back working memory tasks (approximately 20 minutes).

MEG scanning will be conducted using an Elekta Neuromag® TRIUX 306-Channel Magnetometer system (Helsinki, Finland) MEG system, also located at the Centre for Human Psychopharmacology, Swinburne University of Technology. Initial scanning while in a resting state will be conducted in order to collect information as to activity in the DMN. Following this scanning will be conducted whilst participants complete the same in-scanner tasks as used in the fMRI task: verbal episodic memory and N-Back working memory. The two tasks are kept the same across both fMRI and MEG in order for information from the two imaging modalities to be combined into a single comprehensive analysis. MEG scanning provides important complementary information, which is additional to that provided by fMRI. The temporal resolution of MEG is far superior to fMRI; MEG is capable of recording neural oscillations from delta right through to the gamma range (>40 Hz). Although the spatial resolution of MEG is less than that of fMRI, the high number of sensors (approximately 300), together with modern source reconstruction algorithms (for example, beam forming) means that the spatial resolution of MEG is far superior to conventional scalp-recorded EEG [135]. The combination of the two imaging modalities is state-of-the-art and will provide an unparalleled level of analysis of the effects of Lacprodan® PL-20 on memory function.

All primary and secondary outcome measures are displayed in Table 2.

**Table 2:** Summary of PLICAR outcome measures by visit

	**Measures**	**V1**	**V2**	**V3**	**V4**
Screening	Written informed consent	X			
	Demographics	X			
	Medical history	X			
	Inclusion/exclusion criteria	X	X	X	X
	Concomitant medications	X	X	X	X
	Adverse events	X	X	X	X
	Dietary questionnaire	X			
Cognitive	Memory Complaint Questionnaire (MAC-Q)	X			
	Wechsler Memory Scale Revised (WMS-R)	X			
	Raven’s Progressive Matrices (RPM)	X			
	Mini Mental State Exam (MMSE)	X	X	X	X
	Rey Verbal Learning Test (RVLT)	X	X	X	X
	SUCCAB Spatial Working Memory	X	X	X	X
	SUCCAB Contextual Memory	X	X	X	X
	Prospective and Retrospective Memory (PRMQ)	X	X	X	X
	Rapid visual information processing (RVIP)	X	X	X	X
	Serial 3 s and 7 s subtraction	X	X	X	X
	Jensen box task	X	X	X	X
Mood	Beck Depression Inventory (BDI-II)	X			
	Depression, Anxiety and Stress Scale (DASS)	X	X	X	X
	Profile of Mood States (POMS)	X	X	X	X
	Bond-Lader visual analogue mood scales	X	X	X	X
Cardiovascular	Brachial blood pressure		X	X	X
	SphygmoCor (Aortic blood pressure, pulse pressure, PWV)		X	X	X
	Blood velocity (MCA and CCA)		X	X	X
GI microbiota	Intestinal bacteria		X	X	X
Biochemical	Oxidative stress (Glutathione and F2 isoprostanes)		X	X	X
	Inflammation (TNF-α, IL-1β, IL-6 and CRP)		X	X	X
	B Vitamins (B6, B9 and B12)		X	X	X
	Homocysteine (HCy)		X	X	X
	Glucoregulation		X	X	X
	Serum choline		X	X	X
Genetic	Apolipoprotein E and MTHFR		X		
Brain imaging	Structural magnetic resonance imaging (MRI)		X		X
	Diffusion tensor imaging (MRI)		X		X
	Default mode network activation (MEG and MRI)		X		X
	Relaxometry (MRI)		X		X
	Episodic Memory task (functional MRI and MEG)		X		X
	N-Back working memory task (functional MRI and MEG)		X		X

V1, screening/practice session; V2, baseline visit; V3, 9-day visit; V4, 180-day visit; GI, gastrointestinal; SUCCAB, Spatial Working memory and Contextual Memory tasks from the Swinburne University Computerized Cognitive Ageing Battery; PWV, pulse wave velocity; MCA, medial carotid artery; CCA, common carotid artery; CRP, C-reactive protein; MTHFR, Methyltetrahydrofolate reductase; MEG, magnetoencephalography.

### Analysis

The primary analysis will investigate the effect of treatment on all cognitive outcomes from baseline to 180 days, using the groups as randomized (intention to treat). Statistical analyses will be conducted using linear mixed modelling, whereby subject-specific random intercepts and slopes will be fitted to subject data and fixed effects will be fitted to treatment group, time and the treatment × time interaction. On the basis of *APOE* and *MTHFR* genotyping, subgroup analysis will also be conducted in order to investigate the effect of allelic differences on treatment response. Secondary outcome variables will be analysed using similar statistical techniques. Results will be considered statistically significant at an alpha level of *P* <0.05 corrected for multiple comparisons.

Although stratification according to age, intelligence and baseline WMS-R scores may help to explain some of the residual between-group variance unrelated to the treatment effect, further exploration of possible covariates will also be investigated. Baseline correlations between the primary cognitive outcome measures and other baseline variables, including BMI, educational background, diet, CV function, GI microbiota and biochemical parameters, will also be investigated in order to investigate other important covariates. In the event that significant correlations at the *P* <0.05 level are found at baseline then these additional variables will also be controlled for in the primary analysis of cognitive outcomes.

Analysis of functional neuroimaging data (both MEG and fMRI) during episodic memory and N-Back working memory tasks will be conducted using a region of interest (ROI) approach. Using this method, between-group (Lacprodan® PL-20 versus MPC and inert placebo) functional differences in predefined brain regions will be statistically analysed. The ROIs for the episodic memory task will include the medial temporal lobes, the lateral prefrontal cortices, the associative temporal and paretial regions, the cingulate gyrus and the cerebellum. The ROIs that will be analysed in the N-Back working memory task will include the dorsolateral, ventrolateral and medial prefrontal cortex, anterior cingulate, parietal cortex and sensorimotor cortex [136].

### Trial status

The trial is currently recruiting.

## Abbreviations

AAMI: Age-associated memory impairment; AD: Alzheimer’s disease; APOE: Apolipoprotein E; ARCD: Age-related cognitive decline; BDI-II: Beck depression inventory II; BMI: Body mass index; CCA: Common carotid artery; CNS: Central nervous system; CRP: C-reactive protein; V: Cardiovascular; DASS: Depression anxiety and stress scale; DNM: Default mode network; DTI: Diffusion tensor imaging; EEG: Electroencephalography; fMRI: Functional magnetic resonance imaging; GI: Gastrointestinal; GSH: Glutathione; GSSG: Glutathione disulphide; HCy: Homocysteine; L: Interleukin; MAC-Q: Memory complaint questionnaire; MCA: Middle cerebral artery; MEG: Magnetoencephalography; MPC: Milk protein concentrate; MTHFR: Methylenetetrahydrofolate reductase; MSE: Mini mental state Examination; PC: Phosphatidylcholine; PET: Positron emission tomography; PL: Phospholipid; PLICAR: Phospholipid intervention for cognitive ageing reversal; POMS: Profile of mood states; PRMQ: Prospective and retrospective memory questionnaire; PS: Phosphatidylserine; PWV: Pulse wave velocity; ROI: Region of interest; RPM: Raven’s progressive matrices; RVIP: Rapid visual information processing; RVLT: Rey’s verbal learning test; SB: Soybean; SUCCAB: Spatial working memory and contextual memory tasks from the Swinburne university computerized cognitive ageing battery; tHcy: Total homocysteine; TICS-M: Telephone interview for cognitive status – modified; TNF: Tumour necrosis factor; WMS-R: Wechlser memory scale-revised.

## Competing interests

AS and CS receive research funding from the food industry, PF is an employee of Arla Foods. The other authors declare that they have no competing interests.

## Authors’ contributions

AS conceived the study, participated in its design and contributed to drafting the manuscript, DC developed the study design and drafted the manuscript, MH conceived and has responsibility for the fMRI component of the study, WW conceived and has responsibility for the MEG component of the study, CS participated in the study design and contributed to the manuscript, DW participated in the study design and contributed to the manuscript, SG developed the protocol and has responsibility for the microbiota component of the study, PF participated in the study design and contributed to the manuscript. All authors read and approved the final manuscript.
